# Female Nur77-Deficient Mice Show Increased Susceptibility to Diet-Induced Obesity

**DOI:** 10.1371/journal.pone.0053836

**Published:** 2013-01-14

**Authors:** Sonia Perez-Sieira, Gloria Martinez, Begoña Porteiro, Miguel Lopez, Anxo Vidal, Ruben Nogueiras, Carlos Dieguez

**Affiliations:** 1 Department of Physiology, School of Medicine-CIMUS - Instituto de Investigaciones Sanitarias (IDIS), Santiago de Compostela, Spain; 2 CIBER Fisiopatologia de la Obesidad y Nutricion (CIBERobn), San Francisco s/n, Santiago de Compostela (A Coruña), University of Santiago de Compostela, Santiago de Compostela, Spain; Graduate School of Medicine, The University of Tokyo, Japan

## Abstract

Adipose tissue is essential in the regulation of body weight. The key process in fat catabolism and the provision of energy substrate during times of nutrient deprivation or enhanced energy demand is the hydrolysis of triglycerides and the release of fatty acids and glycerol. Nur77 is a member of the NR4A subfamily of nuclear receptors that plays an important metabolic role, modulating hepatic glucose metabolism and lipolysis in muscle. However, its endogenous role on white adipose tissue, as well as the gender dependency of these mechanisms, remains largely unknown. Male and female wild type and Nur77 deficient mice were fed with a high fat diet (45% calories from fat) for 4 months. Mice were analyzed in vivo with the indirect calorimetry system, and tissues were analyzed by real-time PCR and Western blot analysis. Female, but not male Nur77 deficient mice, gained more weight and fat mass when compared to wild type mice fed with high fat diet, which can be explained by decreased energy expenditure. The lack of Nur77 also led to a decreased pHSL/HSL ratio in white adipose tissue and increased expression of CIDEA in brown adipose tissue of female Nur77 deficient mice. Overall, these findings suggest that Nur77 is an important physiological modulator of lipid metabolism in adipose tissue and that there are gender differences in the sensitivity to deletion of the Nur77 signaling. The decreased energy expenditure and the actions of Nur77 on liver, muscle, brown and white adipose tissue contribute to the increased susceptibility to diet-induced obesity in females lacking Nur77.

## Introduction

The orphan nuclear receptor family Nr4a is constituted by three highly homologous members named Nur77 (Nr4a1), Nurr1 (Nr4a2) and Nor1 (Nr4a3) [1]. These nuclear receptors are classified as early response genes that are induced by a diverse range of signals including stress, fatty acids, neurotransmitters, growth factors and cytokines [2]. These orphan nuclear receptors have been implicated in a wide variety of biological actions, including cell cycle regulation, apoptosis, inflammation, carcinogenesis and metabolism [3]–[5]. For instance, overexpression of Nur77 leads to a transcriptional activation of genes involved in inflammation, apoptosis, and cell cycle control [6]. In cancer cells, Nur77 functions in the nucleus as an oncogenic survival factor, but becomes a potent killer when certain death stimuli induce its migration to mitochondria [7].

More recently, it has been demonstrated that Nur77 is an important transcriptional regulator of hepatic and muscle glucose metabolism [8]. In the liver, Nur77 is increased in diabetic mice and its over-expression induces genes involved in gluconeogenesis, stimulates glucose production and elevates blood glucose levels [8]. Moreover, Nur77 deficient mice fed with a high fat diet (HFD) (60% calories from fat) develop hepatic steatosis and impaired insulin sensitivity in both liver and skeletal muscle [9]. In muscle, Nur77 regulates lipolysis, energy expenditure [1], and glucose metabolism [10]. In this sense, Nur77 is expressed at higher levels in glycolytic muscle rather than in oxidative muscle [10], and its overexpression in rat muscle or muscle cells induces the expression of multiple glucose utilization genes [10], [11]. In addition, the stimulation of β-adrenoreceptors induces Nur77 mRNA expression in the C2C12 skeletal muscle cells and elicits skeletal muscle hypertrophy [12].

Although the metabolic actions of Nur77 on liver and muscle seem clear, its potential role on adipose tissue remains controversial. Some *in vitro* studies reported that the expression of NR4a receptors was acutely induced with an adipogenic cocktail in 3T3-L1 preadipocytes and stimulated at long-term after the treatment with PPARγ ligands [13]. On the other hand, in stable 3T3-L1 and 3T3-F442A preadipocyte cell lines that over-express the three nuclear receptors the differentiation was inhibited and this effect could not be restored by PPARγ [14]. Finally, another study indicated that the NR4a family was not required for adipogenesis [15]. *In vivo* studies are limited and there is only one report indicating that Nur77 modulates some of the actions of a α-MSH analog on adipose tissue [16]. For instance, the inhibition of Nur77 blunted the stimulatory effect of a α-MSH analog on important genes involved in inflammation signalling and metabolism in differentiated 3T3-L1 adipocytes [16].

By challenging the mice with a diet of 45% calories from fat we were able to address the physiological function of Nur77 in white and brown adipose tissue (BAT). Additionally, as it is well established that there are sexual differences in the control of energy homeostasis [17], we also uncovered critical gender differences in the Nur77 signaling by using male and female mice lacking Nur77 fed with a HFD of 45% calories from fat. This study demonstrates for first time that female Nur77-deficient mice, but not males, gained more weight and fat due to decreased energy expenditure, an effect that was not detected in males. Although in brown fat we failed to detect any alteration in the expression of enzymes involved in the thermogenic program, female Nur77-deficient mice showed decreased lipolysis in the white adipose tissue. This study indicates that endogenous Nur77 controls the metabolism of white adipose tissue, and together with the metabolic effects of Nur77 on liver and muscle it contributes to increased adiposity.

## Materials and Methods

### Animals

Nur77−/− mice (Lee et al, 1995) were a generous gift by Dr. J. Milbrandt (Washington University School of Medicine in St. Louis). Animals were maintained on a C57BL/6 and 129SvJ hybrid background. Nur77+/− mice were crossed to obtained the experimental animals, wildtype and knockouts, which were always littermates. We used 7–8 per group. Animals were kept in groups of four-five animals under SPF conditions (12∶12 hr light-dark cycle, 22°C) and all experiments were conducted under approval of the Animal Care and Use Committee of the University of Santiago de Compostela. After weaning, mice were fed with a high fat diet (Research Diets 12451; 45% of calories from fat, 4.73 kcal/g, Research Diets, New Brunswick, NJ) during 16 weeks. Animals were decapitated and the tissues were removed rapidly and immediately frozen in liquid nitrogen, and kept at −80°C until their analysis.

### Determination of Body Composition and Energy Balance

After 16 weeks on HFD, whole body composition was measured using NMR imaging (Whole Body Composition Analyzer; EchoMRI, Houston, TX). Animals were monitored in a custom 12-cage indirect calorimetry, food intake and locomotor activity monitoring system (TSE LabMaster, TSE Systems, Germany) as previously described [18], [19]. Mice were acclimated for 48 hr to the test chambers and then were monitored for an additional 48 hr. Data collected from the last 48 hr were used to calculate all parameters for which results are reported, and thereby, cumulative energy expenditure and locomotor activity during 48 h were calculated.

### Quantitative Reverse Transcriptase PCR (qRT-PCR) Analysis

RNA was extracted using Trizol® reagent (Invitrogen) according to the manufacturer's instructions and two micrograms of total RNA were used for each RT reaction and cDNA synthesis was performed using SuperScript™ First-Strand Synthesis System (Invitrogen) and random primers as previously described [20]. Negative control reactions, containing all reagents except the sample were used to ensure specificity of the PCR amplification. For the analysis of gene expression we used real-time reverse-transcription polymerase chain reaction (RT-PCR) analyses performed in a fluorescent temperature cycler (TaqMan®; Applied Biosystems; Foster City, CA, USA) following the manufacturer’s instructions [20], [21]. Five hundred ng of total RNA were used for each RT reaction. The PCR cycling conditions included an initial denaturation at 50°C for 10 min followed by 40 cycles at 95°C for 15 sec; 60°C for 1 min. The oligonucleotide specific primers are indicated in Table 1. For the analysis of the data, the input value of the gene expression was standardized to the 18 S value for the sample group and was expressed compared with the average value for the control group.

**Table 1:** Primers and probes used for gene amplification.

Name	5–3′	Primers
	**FW**	CGGCTACCACATCCAAGGAA
18S	**RV**	GCTGGAATTACCGCGGCT
	**PB**	GACGGCAAGTCTGGTGCCAGCA
	**FW**	TGGGCGGGATGGTCTCTTT
**ACCα1**	**RV**	AGTCGCAGAAGCAGCCCATT
	**PB**	ACCTTTGAAGATTTCGTCAGGATCTTTGATGA
	**FW**	CCCCAAGTGCTGCGATTTC
**β1-adr**	**RV**	AGGTACACGAAGGCCATGATG
	**PB**	TCGTCCGTCGTCTCCTTCTACGTGC
	**FW**	GTGGATCGCTATGTTGCTATCACA
**β2-adr**	**RV**	CACTCGGGCCTTATTCTTGGT
	**PB**	CGCCCTTCAAGTACCAGAGCCTGCT
	**FW**	CACCGCTCAACAGGTTTGATG
**β3-adr**	**RV**	CCCAGAAGTCCTGCAAAAACG
	**PB**	ACGTGAAGGGCCGTGAAGATCCAGC
	**FW**	TCCTCACTGACGCCGACAT
**BMP7**	**RV**	GGTATCGAGGGTGGAAGAATTCT
	**PB**	TCATGAGCTTCGTCAACCTAGTGGAACATG
	**FW**	CCTACGACATCCGATGCACAA
**CIDEA**	**RV**	TCTGTGCAGCATAGGACATAAACC
	**PB**	CTTCAAGGCCGTGTTAAGGAATCTGCTG
	**FW**	TCCAAACGTCACTGCCTAAGCT
**CPT1-B**	**RV**	GGCCGCACAGAATCCAAGT
	**PB**	CGTGCCAGCCACAATTCACCGG
	**FW**	CGCTCCTGGAAAAGGAATCTC
**CPT1-C**	**RV**	CGGGACCACACCAGCAA
	**PB**	CGTGTCTGGAATGACTTT
**CPT1-L**	**FW**	CCCTGGGCATGATTGCAA
	**RV**	GACGCCACTCACGATGTTCTT
	**PB**	CCTAGACACCACTGGCCGCATGTCA
	**FW**	CACTCTGGTACCCAGGACCAATAA
**FGF21**	**RV**	CCCTCAACTTTTCTCTGCCTAGGT
	**PB**	ACAAGAGTAGAGGTGGTGGGCAGAATGCC
	**FW**	CGTGCTGCGGTACAGCC
**INSIG2**	**RV**	GGCTCTCCTAGATGCCTGTCA
	**PB**	CAGCTGTGATTGGACTATTGTACCCCTGCA
	**FW**	GGGAAATGATGTGGCCAGATT
**LPL**	**RV**	CCCTAAGAGGTGGACGTTGTCT
	**PB**	ACTGGATGGAGGAGGAGTTTAACTACCCCC
	**FW**	CGATCACCATATTCCAGGTCAAG
**PGC1α**	**RV**	CGATGTGTGCGGTGTCTGTAGT
**(PPARγ C1α)**	**PB**	AGGTCCCCAGGCAGTAGATCCTCTTCAAGA
	**FW**	CGGTTTCAGAAGTGCCTTGCT
**PPARγ**	**RV**	CGCCAACAGCTTCTCCTTCTC
	**PB**	ATGTCTCACAATGCCATCAGGTTTGGGC
	**FW**	GCGGCTGTTGTCTACCATAAGC
**SREBP1**	**RV**	TGTTGCCATGGAGATAGCATCTC
	**PB**	ACCAGCTGCATGCCATGGGCAAGTA
	**FW**	CGATGTCCATGTACACCAAGGA
**UCP-1**	**RV**	CCCGAGTCGCAGAAAAGAAG
	**PB**	ACCGACGGCCTTTTTCAAAGGGTTTG
	Commercial primers provided by Applied biosystems (Ref.Mm01253292 m1)	
**FASN**		
	
	Commercial primers provided by Applied biosystems (Ref. Mm01266512 m1 )	
**PRDM16**		
	
	Commercial primers provided by Applied biosystems (Ref Rn00565874 ml )	
**UCP-3**		
	

### Western Blot Analysis

Western blots were performed as previously described [19], [22]. Briefly, total protein lysates from liver (20 µg) and epididymal (males) or parametrial (females) WAT (20 µg) were subjected to SDS-PAGE, electrotransferred onto a polyvinylidene difluoride membrane and probed with antibodies against FAS (H-300) (sc-20140), JNK 1/3 (c-17) (sc-474), PTEN (A2B1) (sc-7974) (Santa Cruz Biotechnology, Germany); UCP-1 (Ab10983), HSL/LIPE (ab45422) (Abcam, USA), phospo-HSL (Ser660) (#4126), phospho-SAPK/JNK(thr183/tyr185) (#4671), AKT (#9272), phospho-AKT(Ser473)(#9271) (Cell signaling), beta-actin (A5316) (Sigma-Aldrich, USA). The antibodies dilution was 1∶1000 except UCP-1 (1∶10000). For protein detection we used horseradish peroxidase-conjugated secondary antibodies and chemiluminescence (Thermo Scientific). We used eight mice per group and the protein levels were normalized to β-actin for each sample.

### Glucose and Insulin Tolerance Tests

After 15 weeks on HFD, basal blood glucose levels were measured after an overnight fasting (12 h) with an Accucheck glucometer (Roche). GTT and ITT were done after an intraperitoneal injection of either 2 mg/g D-glucose (Sigma) or 0.75 U/kg insulin (Sigma-Aldrich) as previously described [23]. Area under the curve (AUC) values were determined as previously described [24].

### TG Content in Liver and Muscle

The extraction procedure for tissue TG was adapted from methods described previously [19]. Livers and muscles (aprox 200 mg) were homogenized for 2 min in ice-cold chloroform-methanol (2∶1, vol/vol). TG were extracted during 5-h shaking at room temperature. For phase separation, H_2_SO_4_ was added, samples were centrifuged, and the organic bottom layer was collected. The organic solvent was dried using a Speed Vac and redissolved in chloroform. TG (Randox Laboratories LTD, UK) content of each sample was measured in duplicate after evaporation of the organic solvent using an enzymatic method.

### Hematoxilin/eosin Staining

BAT samples were fixed 24 hour in 10% formalin buffer and then were dehydrated and embedded in paraffin by a standard procedure. Sections of 3 µm were made in a microtome and staining in a standard Hematoxilin/Eosin Alcoholic (BioOptica) procedure as manufacture instructions.

### Levels of Serum Metabolites and Hormones

Serum non-esterified fatty acids (NEFA) concentrations were determined using a kit from Wako (US); triacylglycerol (TG) and cholesterol were determined using a kit from Randox Laboratories (LTD, UK). Serum insulin levels were measured by a previously described RIA [25].

### Data Analysis and Statistics

Values are plotted as the mean ± SEM for each genotype. Statistical analysis was performed using a Mann Whitney U test, comparing male WT vs male Nur77 KO and female WT vs female Nur77 KO. A P value less than 0.05 was considered statistically significant.

## Results

### Female Nur77-null Mice Fed with HFD have Increased Body Weight and Adiposity

**Figure 1:**
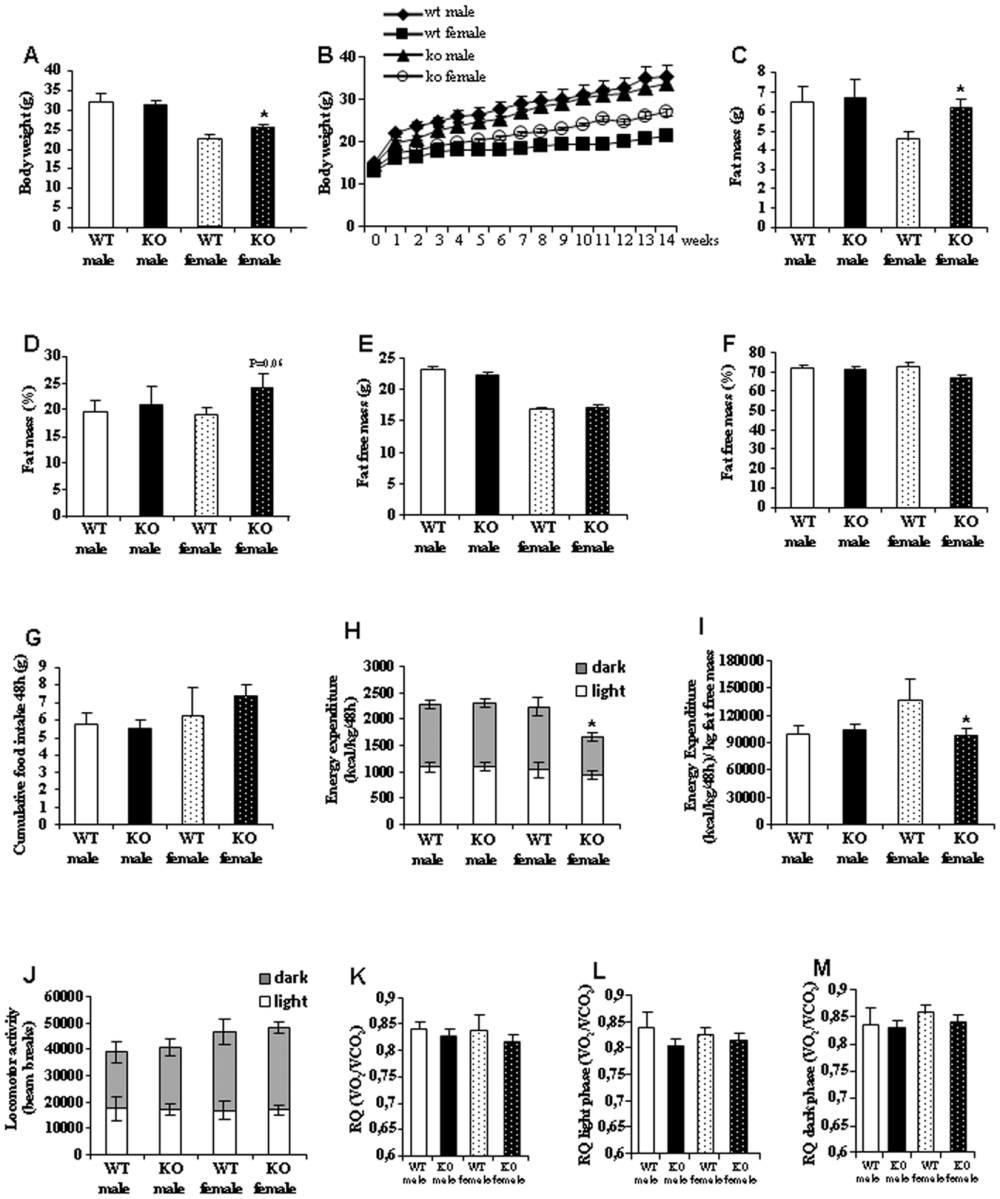
High fat diet changes body weight, adiposity, and energy expenditure in female Nur77 KO mice. (A) Body weight of male and female mice after 16 wk of free access to HFD. (B) Profile of body weight during 14 weeks on HFD. (C) Fat mass in grams. (D) Fat mass as percentage of body weight. (E) Fat-free mass in grams. (F) Fat-free mass as percentage of body weight. (G) Cumulative food intake over 48 hr. (H) 48 hr total energy expenditure determined by indirect calorimetry. Measurements were taken every 45 minutes. Grey squares indicate lights-off. (I) Energy expenditure corrected by kg of fat free mass. (J) Locomotor activity during a 48 hr period. (K) Average of respiratory quotient during 48h. (L) Respiratory quotient during light phase. (M) Respiratory quotient during dark phase. Mice were ∼5 months of age at time of measurement. *p<0.05, n = 7–8 per group.

Age-matched male and female WT and Nur77 KO mice were maintained on HFD from 4 wk of age (45% kcal fat, 4.73 kcal/g) for 16 wk to assess their metabolic phenotypes. Although no differences in body weight were found between male Nur77-deficient and WT mice when they were fed with HFD (Figure 1A–1B), female Nur77 KO mice gained significantly more body weight than WT mice when fed with HFD (Figure 1A–1B). Body composition analysis with quantitative NMR revealed that female Nur77 KO mice fed with HFD during 16 wk accrued more fat mass compared to WT mice (Figure 1C–1D), with no changes in non-fat mass (Figure 1E–1F). However, body composition was similar between male WT and Nur77 KO mice (Figures 1C, 1D, 1E, 1F). Daily HFD intake, measured at the end of the treatment, in Nur77-deficient mice was unchanged relative to WT mice (Figure 1G).

### Deletion of Nur77 Decreases Energy Expenditure in Females Fed with HFD

When fed with HFD, energy expenditure levels were lower in female Nur77-deficient mice than in female WT mice, whereas no changes were detected in males (Figure 1H). The results were similar when energy expenditure was corrected by fat free mass (Figure 1I). In spite of decreased energy expenditure, spontaneous locomotor activity levels remained unchanged between WT and Nur77 KO mice in both sexes (Figure 1J), suggesting that other mechanisms rather than decreased locomotor activity were affecting energy expenditure in female Nur77 KO mice. The respiratory quotient (RQ) remained also unmodified during the circadian cycle (Figure 1K), with no changes during the light (Figure 1L) or dark phase (Figure 1M) suggesting that the lack of Nur77 does not modulate nutrient partitioning.

### Female Nur77 KO Mice Fed with HFD have Increased Hepatic Fat Storage

**Figure 2:**
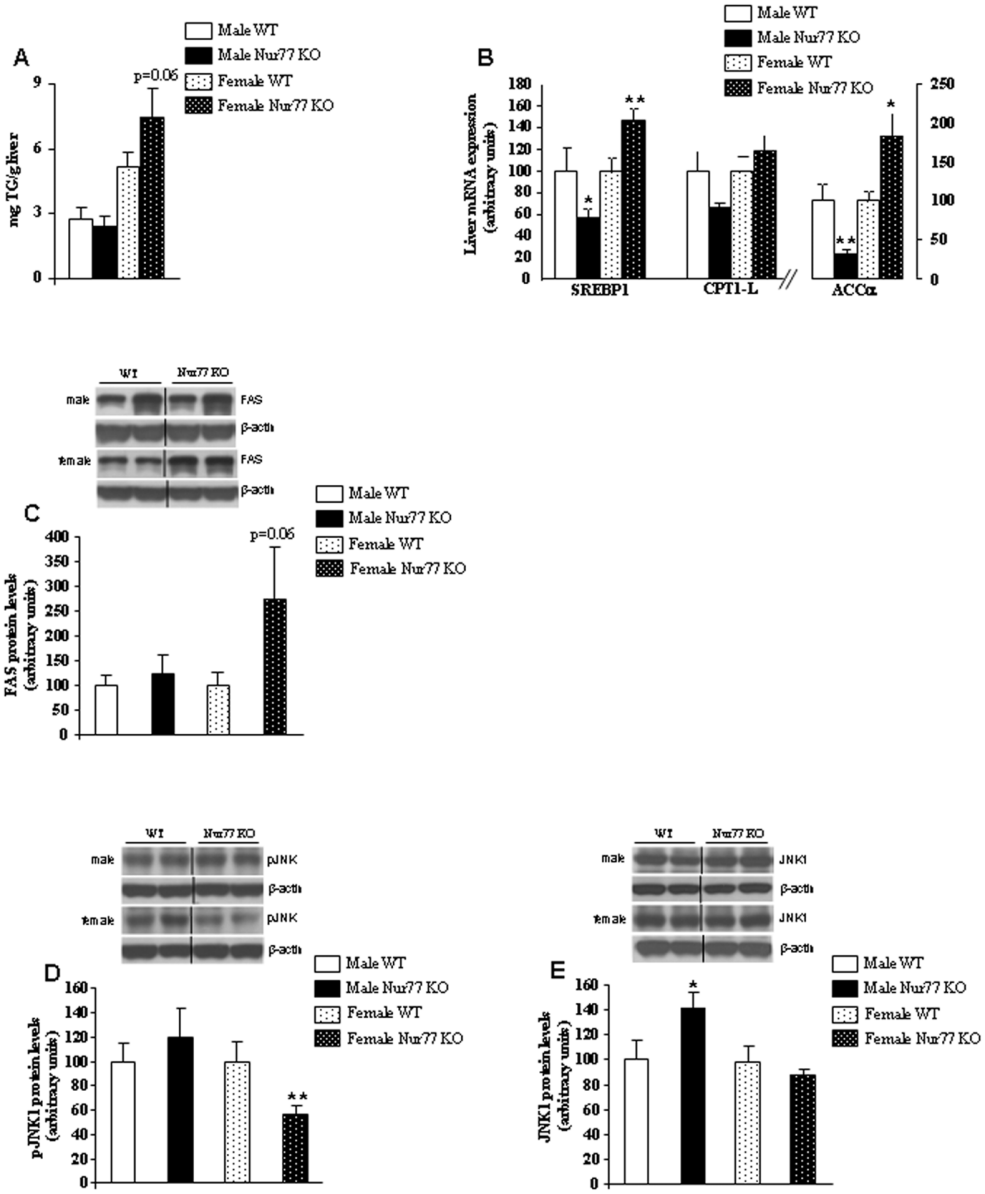
Effect of Nur77 deficiency in the liver of mice fed with HFD. (A) Total liver triglyceride (TG) content after HFD exposure. mRNA expression of SREBP1, CPT1-L and ACCα (Figure 2B) in the liver of WT and Nur77 KO mice. Representative western blot of protein levels of (C) FAS, (D) pJNK1 and (E) JNK1 in the liver of WT and Nur77 KO mice. Dividing lines indicate splicings in the figure. *p<0.05, **p<0.01, n = 7–8 per group.

In agreement with increased body weight and fat mass gain, we found increased total hepatic TG content in female Nur77-deficient mice fed with HFD in comparison to WT mice (Figure 2A). Higher liver TG levels were consistent with an up-regulation of the mRNA expression of SREBP1 and ACCα (Figure 2B) and protein levels of FAS, a lipogenic marker, (Figure 2C) and with decreased levels of pJNK1 (Figure 2D), a kinase strongly implicated in glucose and lipid metabolism [26] in female Nur77-deficient mice fed with HFD in comparison to WT mice. However, no changes were detected in total JNK1 protein levels in females (Figure 2E). Taken together, these data suggest that the lack of Nur77 favours lipid storage in the liver of female Nur77-deficient mice fed with HFD.

### Female Nur77 KO Mice Fed with HFD Show Decreased Lipolysis in White Adipose Tissue

**Figure 3:**
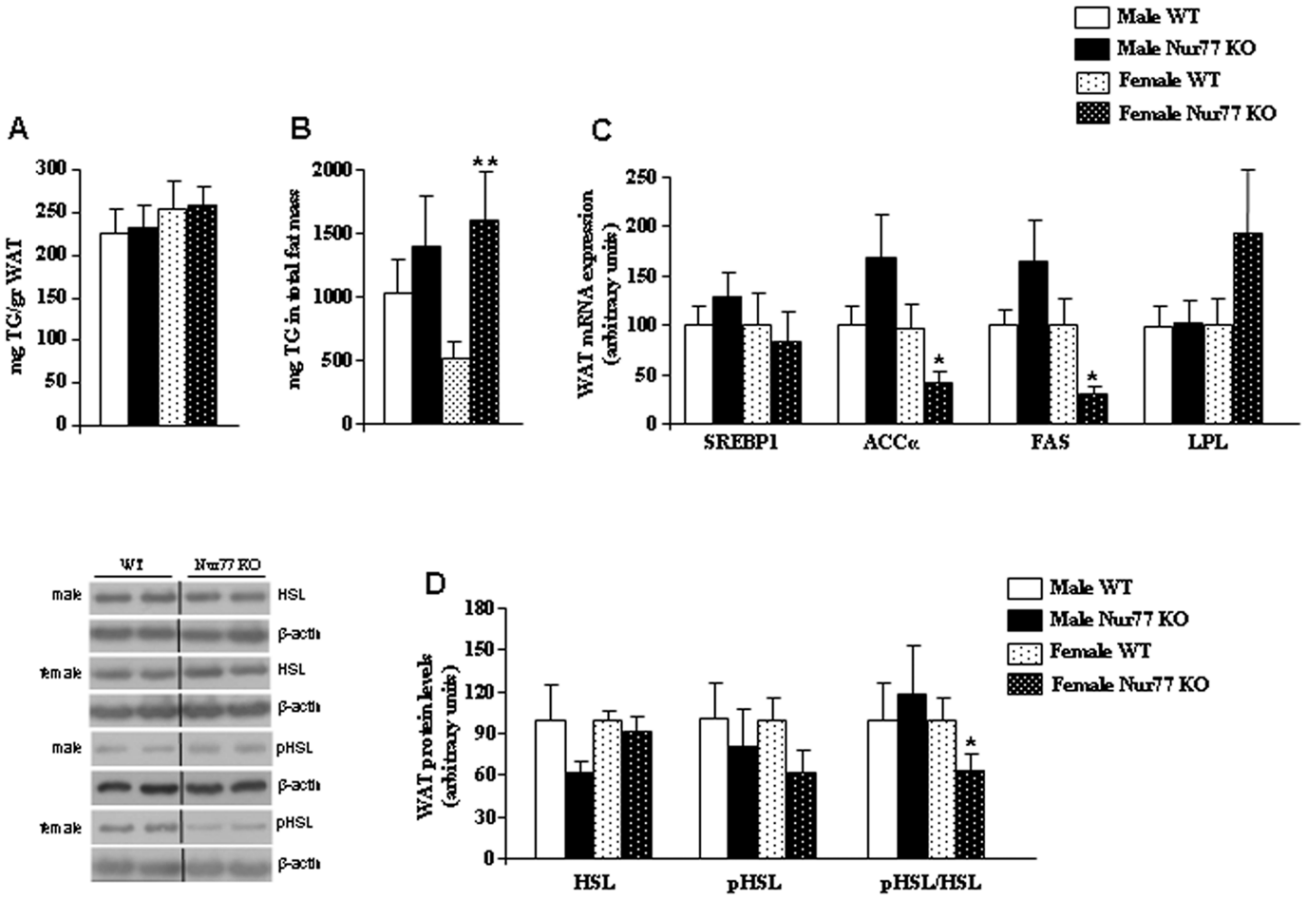
Lipid metabolism in gonadal white adipose tissue in Nur77 KO mice fed with HFD. (A) mg TG content per gram of WAT. (B) mg TG content in the total amount of WAT. (C) mRNA expression of enzymes involved in lipid storage: SREBP1, ACCα, FAS, and LPL in white adipose tissue after 16 wk of HFD. (D) Protein levels of HSL, pHSL, and the ratio pHSL/HSL. Dividing lines indicate splicings in the figure. *p<0.05, n = 7–8 per group.

Having shown that the amount of fat mass was higher in female Nur77-deficient mice fed with HFD when compared to their WT controls, we first measured the TG content per gram of fat and found no differences between WT and Nur77 KO mice (Figure 3A). However, when we calculated the TG content in the total amount of fat mass we found a significant increase in female Nur77 KO in comparison to their WT controls (Figure 3B). Then, we investigated the molecular pathways mediating adipocyte lipid metabolism. For this, we used epididymal fat from males and parametrial fat from females. We found a significant decrease in the mRNA expression of ACCα and FAS (Figure 3C), with no changes in SREBP1 or LPL gene levels in the parametrial WAT of female Nur77 KO mice fed with HFD (Figure 3C). However, we failed to detect any modification in the parametrial WAT mRNA expression of CPT1, INSIG2, β1-AR, β2-AR, β3-AR, or PGC1α between Nur77-deficient mice and WT mice fed with HFD (data not shown). To further explore underlying mechanisms involved in lipid catabolism, we examined the protein levels of hormone-sensitive lipase (HSL), a key enzyme in fatty acid mobilization and lipolysis in the WAT. Although Nur77 deficiency did not change total of phosphorylated levels of HSL, the ratio pHSL/HSL was decreased in the parametrial WAT of female Nur77-deficient mice (Figure 3D). Overall, these results indicate that lipolysis is inhibited in the WAT of female Nur77 deficient mice fed with HFD.

### Deletion of Nur77 in Females Increases CIDEA Levels in BAT

**Figure 4:**
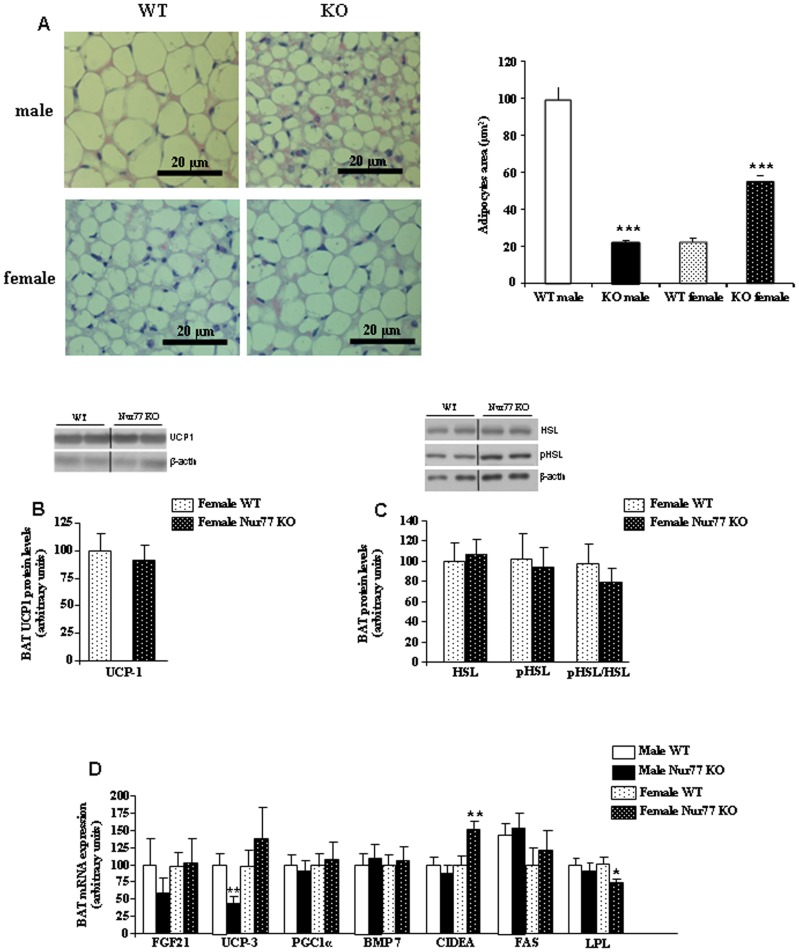
BAT metabolism in Nur77 KO mice fed with HFD. (A) Representative pictures of BAT histology and quantification the size of brown adipocytes after 16 wk of HFD. (B) Protein levels of UCP1 in BAT of female WT and Nur77 KO mice after 16 wk of HFD. (C) Protein levels of HSL, pHSL, and the ratio pHSL/HSL in BAT of female WT and Nur77 KO mice after 16 wk of HFD. Dividing lines indicate splicings in the figure. (D) mRNA expression of factors involved in thermogenesis and lipid metabolism: FGF21, UCP3, PGC1α, BMP7, CIDEA, FAS, and LPL in BAT of male and female WT and Nur77 KO mice after 16 wk of HFD. **p<0.01, n = 7–8 per group.

To investigate the potential mechanism responsible for decreased energy expenditure in female Nur77 KO mice, we analyzed the BAT. Brown adipocytes from females Nur77 KO were bigger than those of female WT mice fed a HFD (Figure 4A). Contrary, brown adipocytes from males Nur77 KO were smaller than those of male WT fed a HFD (Figure 4A). We next measured the expression of several thermogenic markers in BAT. There were no differences in the protein levels of UCP1 between females Nur77 KO and WT (Figure 4B) or the expression of HSL and pHSL (Figure 4C). Consistently, the mRNA expression of other factors involved in the thermogenic program such as UCP3, FGF21, PGC1α or BMP7 was also unaltered in female Nur77 deficient mice (Figure 4D). However, LPL mRNA expression was decreased in the BAT of female Nur77 KO mice (Figure 4D). We also assessed the levels of CIDEA, since it is known that mice lacking CIDEA had higher metabolic rate, lipolysis in BAT and core body temperature when subjected to cold treatment 27]. We found that mRNA levels of CIDEA were increased in the BAT of female Nur77KO mice (Figure 4D). In males Nur77 KO we only detected a significant decrease in UCP-3 gene expression when compared to their WT controls (Figure 4D).

### Female Nur77 KO Mice have Normal Serum NEFAs, TG, Cholesterol and Insulin Levels but Increased Leptin and Basal Glucose Levels

We failed to detect any significant change in circulating levels of non-esterified fatty acids (NEFAs), TG, cholesterol and insulin between WT and Nur77 KO mice maintained on HFD (Table 2). However, we found a significant increase in leptin and basal (overnight fasting) glucose levels in female Nur77 deficient mice in comparison to female WT mice (Table 2).

**Table 2:** Circulating parameters of non-esterified fatty acids (NEFAs), triglycerides (TG), cholesterol, insulin, leptin and basal glucose in WT and Nur77 KO mice after 16 wk of HFD.

	WT males	KO males	WT females	KO females
Initial body weight (g)	15.00±0.42	14.80±0.98	12.93±0.23	13.56±0.33
NEFAs (mg/dl)	77.34±9.98	76.49±20.74	36.88±4.27	41.99±2.23
TG (mg/dl)	138.17±21.35	117.66±23.04	80.66±11.31	74.40±3.60
Cholesterol (mg/dl)	363.43±14.15	342.77±13.08	246.52±18.21	253.33±18.96
Insulin (ng/ml)	1.71±0.41	2.44±0.92	0.55±0.06	0.79±0.12
Leptin (ng/ml)	9.87±2.05	15.59±4.24	3.99±1.41	9.95±1.96*
Basal Glucose (mg/dl)	93.60±11.47	97.83±12.94	53.00±10.79	80.60±5.30 *

n = 7–8 per group. *p<0.05.

### TG Content and Insulin Signalling in Muscle

**Figure 5:**
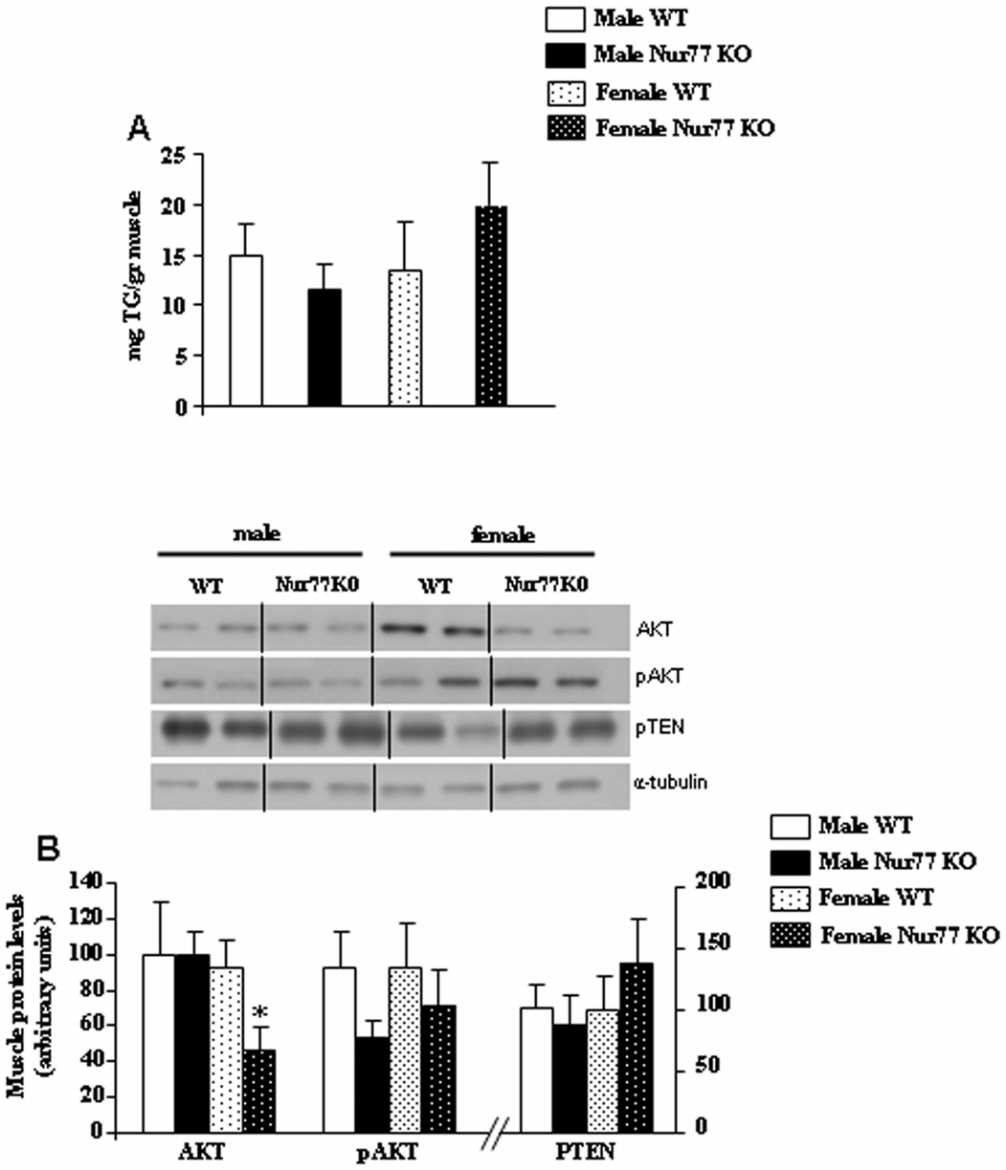
Effect of Nur77 deficiency in the muscle of mice fed with HFD. (A) Total muscle triglyceride (TG) content after HFD exposure. (B) Representative western blot of protein levels of AKT, pAKT and PTEN in the muscle of male and female WT and Nur77 KO mice. Dividing lines indicate splicings in the figure. *p<0.05, **p<0.01, n = 7–8 per group.

Muscle TG content was unchanged between male WT and Nur77 KO mice, whereas it was slightly, but not significantly, increased in female Nur77 deficient mice when compared to their WT controls (Figure 5A). Next, we assessed some key factors mediating insulin signalling and found that protein levels of AKT were decreased in the muscle of female mice lacking Nur77, whereas no changes were detected for pAKT or PTEN (Figure 5B).

### Glucose Homeostasis in Nur77-deficient Mice fed with High Fat Diet

**Figure 6:**
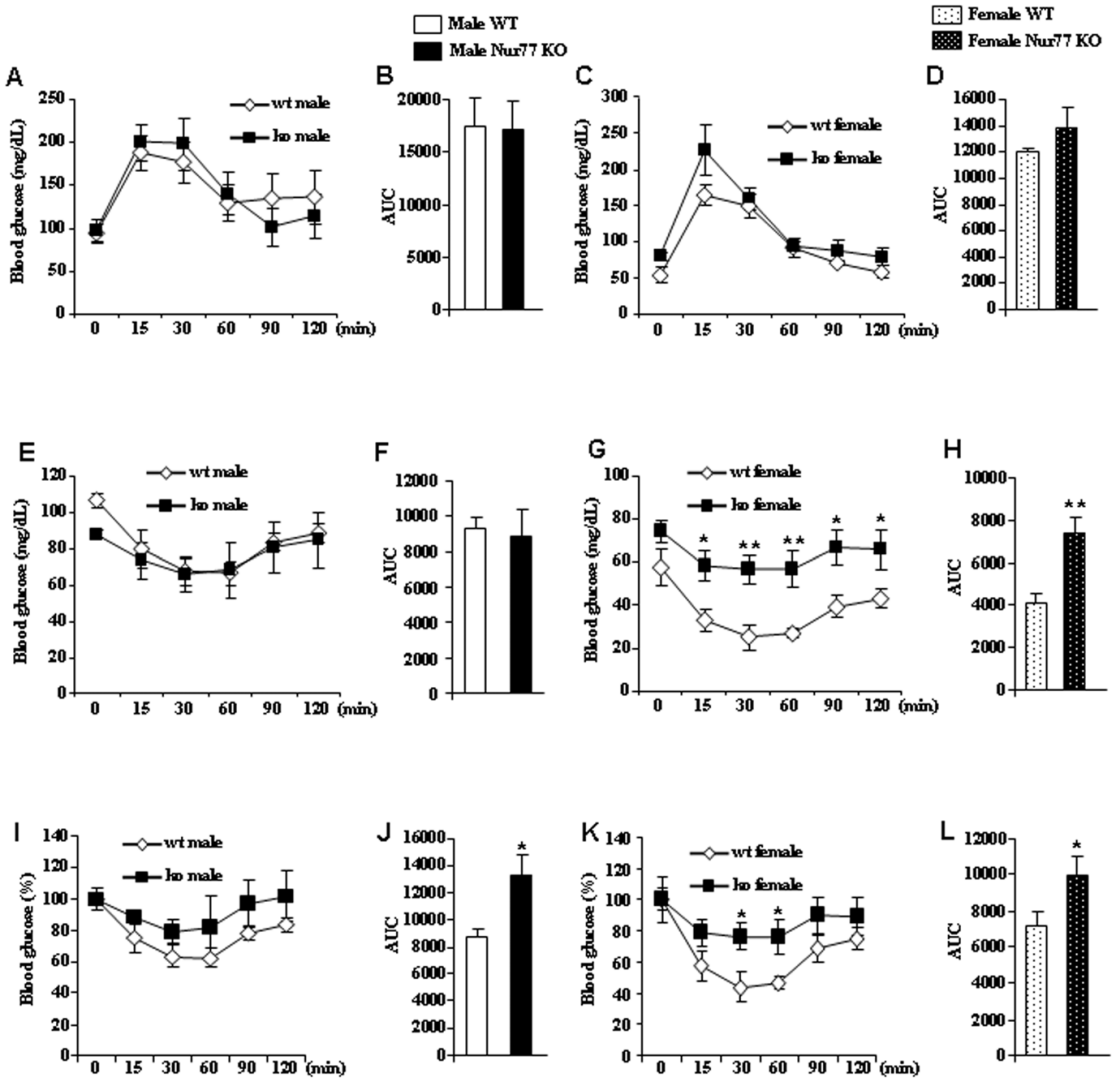
Glucose tolerance and insulin sensitivity in Nur77 KO mice. (A) Intraperitoneal glucose tolerance test (ipGTT) in males after 16 wk of HFD. (B) Respective area under the curve (AUC). (C) ipGTT in females after 16 wk of HFD. (D) Respective AUC. (E) Insulin tolerance test (ITT) in males after 16 wk of HFD. (F) Respective AUC. (G) ITT in females after 16 wk of HFD. (H) Respective AUC. (I) Delta glycemia during an ITT in males after 16 wk of HFD. (J) Respective AUC. (K) Delta glycemia during an ITT in females after 16 wk of HFD. (L) Respective AUC. *p<0.05, **p<0.01, n = 7–8 per group.

We assessed key parameters of glucose homeostasis in WT and Nur77 KO mice. This analysis revealed unaltered fasting blood glucose concentrations in male Nur77 null mice compared to controls fed with high fat diet (Table 2). However, female Nur77 Ko mice showed increased fasting serum glucose levels in comparison to WT females (Table 2). When we subjected Nur77 KO mice fed with high fat diet to intraperitoneal glucose tolerance tests (ipGTT), we did not find any alteration in glucose tolerance in males (Figure 6A–6B) or females (Figure 6C–6D), indicating that female Nur77 KO mice did not show differences in glucose tolerance.

Furthermore, we subjected Nur77 deficient mice to insulin tolerance tests (ITT) and found unaltered blood glucose levels in male Nur77 KO fed with HFD in comparison to WT males (Figure 6E–6F). However, we observed a marked insulin resistance in female Nur77 KO mice in comparison to female WT mice fed with HFD (Figure 6G–6H). Interestingly, when we calculated glucose as delta glycemia we found that both male (Figure 6I–6J) and female (Figure 6K–6L) mice lacking Nur77 were more insulin resistant than their WT controls.

## Discussion

Nur77 is involved in the regulation of several biological actions, including metabolism. In particular, Nur77 is a transcriptional regulator of glucose metabolism in liver and skeletal muscle [8]–[10], [12]. *In vitro* studies have also indicated that Nur77 is involved in adipogenesis [14], [16]. However, there have been no *in vivo* studies identifying the role of Nur77 in adipose tissue. Using a HFD of 45% calories from fat, in the present work, we demonstrate that female mice lacking Nur77 fed with a HFD gained significantly more body weight than WT controls. The decreased energy expenditure was consistent with increased body weight and fat mass in Nur77 KO mice after HFD. At molecular level, we found alterations in lipid metabolism in female, but not male mice. More specifically, our findings suggest that the deficiency of Nur77 decreased adipose tissue lipolysis in females in comparison to their littermates fed with HFD.

In addition to the storage of lipids function, WAT plays a crucial role in the maintenance of energy homeostasis. Furthermore, BAT has emerged as an active organ regulating thermogenesis not only in the newborn but also in adult humans [28]–[30]. It is well established that both WAT and BAT exert relevant effects on insulin sensitivity. Thus, in the work presented here, we have investigated the role of Nur77 in these tissues. We found a decreased pHSL/HSL ratio in WAT, which suggest a decreased lipolysis. This is consistent with the effect of Nur77 in skeletal muscle cells, where the attenuation of Nur77 expression resulted in decreased lipolysis [1]. These results are somewhat in contrast with the ones observed in humans, since Nur77 is up-regulated in the WAT of extreme obese patients, whereas Nur77 expression is normalized after fat loss [31]. Taken together, these findings suggest that the increased expression of Nur77 in the WAT of obese patients could be a compensatory mechanism in order to limit adipose tissue expansion.

In BAT, the expression of Nur77 was induced in cold-exposed mice and that Nur77 is a cold-induced negative regulator of UCP1 [32]. However, mice lacking this receptor do not show any alteration in non-shivering thermogenesis, suggesting that there is a compensatory mechanism of the other members of the family [32]. Our present data are in agreement with this, and we failed to detect any significant change in the expression of genes controlling the thermogenic program in BAT of Nur77 deficient mice. However, we found increased levels of CIDEA in the BAT of female Nur77 KO mice compared to their littermates fed with HFD. Notably, CIDEA is an important inhibitor of lipolysis and CIDEA deficient mice are lean and resistant to diet-induced obesity and diabetes [27]. Thus, our findings suggest that increased levels of CIDEA might contribute to decrease lipolysis, whereas the reduced expression of LPL in the BAT of female Nur77 KO mice suggests a lower lipid uptake in BAT of female Nur77 KO mice. The enlarged size of brown adipocytes from female Nur77 deficient mice in comparison to female WT mice might be likely explained by decreased lipolysis in brown adipocytes.

An intriguing question of this study is the gender dimorphism observed in the metabolic phenotype of mice lacking Nur77. There is a wealth of clinical and experimental data demonstrating that sex steroids and insulin interact in their effects on several tissues [33]. The deficiency of estrogens or its receptors is associated with increased adiposity, in particular in visceral fat, which impairs insulin sensitivity [17], [34]. Moreover, restoration of estrogens levels in ovariectomized mice blunts the body weight gain. Estrogens also affect energy expenditure and lipid metabolism, for instance ERα-deficient mice showed lower energy expenditure [34], and ERα directly activates lipolysis [35]. Thus, it is tempting to speculate that the phenotype observed in female Nur77 deficient mice might be caused by alterations in the estrogen receptor signalling, a hypothesis that will require further investigation.

A previous report has described that male mice lacking Nur77 gained more weight and were more insulin resistant than WT mice [9]. Our findings show that female, but not male Nur77-deficient mice gained more weight when fed a HFD. However, both female and male Nur77-deficient mice, showed increased susceptibility to diet-induced obesity and insulin resistance. The observed insulin resistance of female and male Nur77-deficient mice is consistent to the tendency of increased fasting insulin levels in Nur77 deficient mice.

The discrepancies on body weight gain between the two studies might be explained by differences in the methodological approaches. We fed the mice with a HFD of 45% calories from fat, whereas the previous work used 60% calories from fat [9]. Indeed, the use of diets with a different percentage of fat causes different alterations in the content of TG, cholesterol, etc [36]. Thus, this important variable allows to detect different actions of one gene using the same animal model. By challenging the mice with a diet with a lower fat content we were able to uncover the gender differences here reported. In support of this it is noteworthy that although we failed to find any difference in males, the metabolic phenotype of female Nur77-deficient mice resembles to the one reported in male Nur77-deficient mice. In any event, our data suggest that the increased body weight and fat content in mice lacking Nur77 fed with HFD can be caused by decreased energy expenditure. Moreover, this is accompanied by increased triglyceride content in the liver and by a marked impairment in insulin sensitivity. Additionally, we found that phosphorylated levels of JNK1 are decreased in the liver of female Nur77-deficient mice fed with HFD. Since it was demonstrated that ablation of JNK1 in hepatocytes induces insulin resistance and hepatic steatosis [37], our data suggest that besides the reported mechanisms triggering fat accumulation in the liver [9], JNK1 is also mediating the effects of Nur77 on hepatic metabolism. Thus, our current study supports previous findings and overall, it can be concluded that the endogenous Nur77 plays an important role in the control of energy homeostasis and glucose metabolism.

In addition to its hepatic actions, when Nur77 deficient mice were fed with a HFD of 60%, it has been also reported that depletion of Nur77 increased intramuscular lipid content, and impaired fatty acid oxidation and insulin resistance in muscle [9], [10]. Similarly, in the present work we found that a HFD of 45% calories from fat, induced a moderated increase of TG content in muscle and inhibited AKT protein levels in females lacking Nur77, which might partially explain the impaired insulin sensitivity observed in these mice.

Overall, it is likely that increased adiposity in female nur77 deficient mice reflects the endpoint of changes in systemic metabolic effects relating to the action of Nur77 in several important metabolic tissues, including not only skeletal muscle [9], [10] and liver [8], but also white and brown fat.

In summary, this study demonstrates that: a) in addition to its known metabolic roles on liver and skeletal muscle, Nur77 is also an important physiological modulator of lipid metabolism in adipose tissue; and b) the choice of a HFD of 45% calories from fat, demonstrates that there are gender differences in the sensitivity to deletion of the Nur77 signaling. The decreased energy expenditure and the metabolic alteration in liver, muscle and adipose tissue favour the increased susceptibility to diet-induced obesity in mice lacking Nur77. These data suggest that the activation of Nur77 in metabolic peripheral tissues might be effective in the treatment of obesity and its associated disorders.

## References

[1] Maxwell MA, Cleasby ME, Harding A, Stark A, Cooney GJ, et al (2005) Nur77 regulates lipolysis in skeletal muscle cells. Evidence for cross-talk between the beta-adrenergic and an orphan nuclear hormone receptor pathway. J Biol Chem 280: 12573–12584. 15640143 10.1074/jbc.M409580200

[2] Maxwell MA, Muscat GE (2006) The NR4A subgroup: immediate early response genes with pleiotropic physiological roles. Nucl Recept Signal 4: e002. 16604165 10.1621/nrs.04002 PMC1402209

[3] Lee SL, Wesselschmidt RL, Linette GP, Kanagawa O, Russell JH, et al (1995) Unimpaired thymic and peripheral T cell death in mice lacking the nuclear receptor NGFI-B (Nur77). Science 269: 532–535. 7624775 10.1126/science.7624775

[4] Mullican SE, Zhang S, Konopleva M, Ruvolo V, Andreeff M, et al (2007) Abrogation of nuclear receptors Nr4a3 and Nr4a1 leads to development of acute myeloid leukemia. Nat Med 13: 730–735. 17515897 10.1038/nm1579

[5] Pearen MA, Muscat GE (2010) Minireview: Nuclear hormone receptor 4A signaling: implications for metabolic disease. Mol Endocrinol 24: 1891–1903. 20392876 10.1210/me.2010-0015 PMC5417389

[6] Pei L, Castrillo A, Tontonoz P (2006) Regulation of macrophage inflammatory gene expression by the orphan nuclear receptor Nur77. Mol Endocrinol 20: 786–794. 16339277 10.1210/me.2005-0331

[7] Moll UM, Marchenko N, Zhang XK (2006) p53 and Nur77/TR3 - transcription factors that directly target mitochondria for cell death induction. Oncogene 25: 4725–4743. 16892086 10.1038/sj.onc.1209601

[8] Pei L, Waki H, Vaitheesvaran B, Wilpitz DC, Kurland IJ, et al (2006) NR4A orphan nuclear receptors are transcriptional regulators of hepatic glucose metabolism. Nat Med 12: 1048–1055. 16906154 10.1038/nm1471

[9] Chao LC, Wroblewski K, Zhang Z, Pei L, Vergnes L, et al (2009) Insulin resistance and altered systemic glucose metabolism in mice lacking Nur77. Diabetes 58: 2788–2796. 19741162 10.2337/db09-0763 PMC2780886

[10] Chao LC, Zhang Z, Pei L, Saito T, Tontonoz P, et al (2007) Nur77 coordinately regulates expression of genes linked to glucose metabolism in skeletal muscle. Mol Endocrinol 21: 2152–2163. 17550977 10.1210/me.2007-0169 PMC2602962

[11] Kanzleiter T, Preston E, Wilks D, Ho B, Benrick A, et al (2010) Overexpression of the orphan receptor Nur77 alters glucose metabolism in rat muscle cells and rat muscle in vivo. Diabetologia 53: 1174–1183. 20217038 10.1007/s00125-010-1703-2

[12] Pearen MA, Ryall JG, Maxwell MA, Ohkura N, Lynch GS, et al (2006) The orphan nuclear receptor, NOR-1, is a target of beta-adrenergic signaling in skeletal muscle. Endocrinology 147: 5217–5227. 16901967 10.1210/en.2006-0447

[13] Fu M, Sun T, Bookout AL, Downes M, Yu RT, et al (2005) A Nuclear Receptor Atlas: 3T3-L1 adipogenesis. Mol Endocrinol 19: 2437–2450. 16051663 10.1210/me.2004-0539

[14] Chao LC, Bensinger SJ, Villanueva CJ, Wroblewski K, Tontonoz P (2008) Inhibition of adipocyte differentiation by Nur77, Nurr1, and Nor1. Mol Endocrinol 22: 2596–2608. 18945812 10.1210/me.2008-0161 PMC2610364

[15] Au WS, Payne VA, O'Rahilly S, Rochford JJ (2008) The NR4A family of orphan nuclear receptors are not required for adipogenesis. Int J Obes (Lond) 32: 388–392. 18071346 10.1038/sj.ijo.0803763

[16] Wang SC, Myers SA, Eriksson NA, Fitzsimmons RL, Muscat GE (2011) Nr4a1 siRNA expression attenuates alpha-MSH regulated gene expression in 3T3-L1 adipocytes. Mol Endocrinol 25: 291–306. 21239615 10.1210/me.2010-0231 PMC5417310

[17] Shi H, Seeley RJ, Clegg DJ (2009) Sexual differences in the control of energy homeostasis. Front Neuroendocrinol 30: 396–404. 19341761 10.1016/j.yfrne.2009.03.004 PMC4517605

[18] Czyzyk TA, Nogueiras R, Lockwood JF, McKinzie JH, Coskun T, et al (2010) kappa-Opioid receptors control the metabolic response to a high-energy diet in mice. FASEB J 24: 1151–1159. 19917675 10.1096/fj.09-143610 PMC2845433

[19] Nogueiras R, Perez-Tilve D, Veyrat-Durebex C, Morgan DA, Varela L, et al (2009) Direct control of peripheral lipid deposition by CNS GLP-1 receptor signaling is mediated by the sympathetic nervous system and blunted in diet-induced obesity. J Neurosci 29: 5916–5925. 19420258 10.1523/JNEUROSCI.5977-08.2009 PMC6665241

[20] Gonzalez CR, Caminos JE, Vazquez MJ, Garces MF, Cepeda LA, et al (2009) Regulation of visceral adipose tissue-derived serine protease inhibitor by nutritional status, metformin, gender and pituitary factors in rat white adipose tissue. J Physiol 587: 3741–3750. 19470778 10.1113/jphysiol.2009.172510 PMC2742295

[21] Vazquez MJ, Gonzalez CR, Varela L, Lage R, Tovar S, et al (2008) Central resistin regulates hypothalamic and peripheral lipid metabolism in a nutritional-dependent fashion. Endocrinology 149: 4534–4543. 18499762 10.1210/en.2007-1708

[22] Velasquez DA, Martinez G, Romero A, Vazquez MJ, Boit KD, et al (2011) The central Sirtuin 1/p53 pathway is essential for the orexigenic action of ghrelin. Diabetes 60: 1177–1185. 21386086 10.2337/db10-0802 PMC3064091

[23] Lee SJ, Kim JY, Nogueiras R, Linares JF, Perez-Tilve D, et al (2010) PKCzeta-regulated inflammation in the nonhematopoietic compartment is critical for obesity-induced glucose intolerance. Cell Metab 12: 65–77. 20620996 10.1016/j.cmet.2010.05.003 PMC2907185

[24] Czyzyk TA, Nogueiras R, Lockwood JF, McKinzie JH, Coskun T, et al (2010) kappa-Opioid receptors control the metabolic response to a high-energy diet in mice. FASEB J 24: 1151–1159. 19917675 10.1096/fj.09-143610 PMC2845433

[25] Lopez M, Lage R, Saha AK, Perez-Tilve D, Vazquez MJ, et al (2008) Hypothalamic fatty acid metabolism mediates the orexigenic action of ghrelin. Cell Metab 7: 389–399. 18460330 10.1016/j.cmet.2008.03.006

[26] Sabio G, Davis RJ (2010) cJun NH2-terminal kinase 1 (JNK1): roles in metabolic regulation of insulin resistance. Trends Biochem Sci 35: 490–496. 20452774 10.1016/j.tibs.2010.04.004 PMC2975251

[27] Zhou Z, Yon Toh S, Chen Z, Guo K, Ng CP, et al (2003) Cidea-deficient mice have lean phenotype and are resistant to obesity. Nat Genet 35: 49–56. 10.1038/ng1225 12910269

[28] van Marken Lichtenbelt WD, Vanhommerig JW, Smulders NM, Drossaerts JM, Kemerink GJ, et al (2009) Cold-activated brown adipose tissue in healthy men. N Engl J Med 360: 1500–1508. 19357405 10.1056/NEJMoa0808718

[29] Cypess AM, Lehman S, Williams G, Tal I, Rodman D, et al (2009) Identification and importance of brown adipose tissue in adult humans. N Engl J Med 360: 1509–1517. 19357406 10.1056/NEJMoa0810780 PMC2859951

[30] Virtanen KA, Lidell ME, Orava J, Heglind M, Westergren R, et al (2009) Functional brown adipose tissue in healthy adults. N Engl J Med 360: 1518–1525. 19357407 10.1056/NEJMoa0808949

[31] Veum VL, Dankel SN, Gjerde J, Nielsen HJ, Solsvik MH, et al.. (2011) The nuclear receptors NUR77, NURR1 and NOR1 in obesity and during fat loss. Int J Obes (Lond). 10.1038/ijo.2011.240 22143616

[32] Kanzleiter T, Schneider T, Walter I, Bolze F, Eickhorst C, et al (2005) Evidence for Nr4a1 as a cold-induced effector of brown fat thermogenesis. Physiol Genomics 24: 37–44. 16219868 10.1152/physiolgenomics.00204.2005

[33] Livingstone C, Collison M (2002) Sex steroids and insulin resistance. Clin Sci (Lond) 102: 151–166. 11834135 10.1042/cs1020151

[34] Heine PA, Taylor JA, Iwamoto GA, Lubahn DB, Cooke PS (2000) Increased adipose tissue in male and female estrogen receptor-alpha knockout mice. Proc Natl Acad Sci U S A 97: 12729–12734. 11070086 10.1073/pnas.97.23.12729 PMC18832

[35] Barros RP, Gustafsson JA (2011) Estrogen receptors and the metabolic network. Cell Metab 14: 289–299. 21907136 10.1016/j.cmet.2011.08.005

[36] Prior RL, Wu X, Gu L, Hager T, Hager A, et al (2009) Purified berry anthocyanins but not whole berries normalize lipid parameters in mice fed an obesogenic high fat diet. Mol Nutr Food Res 53: 1406–1418. 19743407 10.1002/mnfr.200900026

[37] Sabio G, Cavanagh-Kyros J, Ko HJ, Jung DY, Gray S, et al (2009) Prevention of steatosis by hepatic JNK1. Cell Metab 10: 491–498. 19945406 10.1016/j.cmet.2009.09.007 PMC2804105

